# Kugel’s Artery in Coronary Computed Tomography Angiography in a Patient with Left Main Subtotal Stenosis

**DOI:** 10.3390/diagnostics14192142

**Published:** 2024-09-26

**Authors:** Paweł Gać, Agnieszka Głuszczyk, Jakub Plizga, Wiktoria Bińczyk, Olgierd Dróżdż, Rafał Poręba

**Affiliations:** 1 Centre of Diagnostic Imaging, 4th Military Hospital, Weigla 5, 50-981 Wrocław, Poland; 2 Department of Environmental Health, Occupational Medicine and Epidemiology, Wroclaw Medical University, Mikulicza-Radeckiego 7, 50-345 Wrocław, Poland; 3 Department of Angiology and Internal Medicine, Wroclaw Medical University, Borowska 213, 50-556 Wrocław, Poland

**Keywords:** Kugel’s artery, coronary computed tomography angiography, left main stenosis, left circumflex, right coronary artery

## Abstract

The artery of Kugel is defined as a rare anatomical variant of the coronary arteries. It consists of an anastomotic connection between the branches of the right coronary artery (RCA) and the left circumflex artery (LCx). In patients with coronary artery occlusion, the presence of this connection bypassing the area of the occluded vessel may be a factor that prevents significant necrosis of a specific area of the myocardium. Most cases are detected by invasive coronary angiography. We present images from coronary computed tomography angiography (CCTA), which documented the existence of Kugel’s artery in a 67-year-old patient with subtotal stenosis of the left main artery. The presented images clearly indicate that CCTA can visualize the existing rescue collateral circulation in patients with significant coronary artery disease.

The artery of Kugel is defined as a rare anatomical variant of the coronary arteries. It consists of an anastomotic connection between the branches of the right coronary artery (RCA) and the left circumflex artery (LCx). In most cases, it anastomoses directly or through its branches with the distal part of the RCA; in the rest of the cases, this artery formed connections with the proximal part of the RCA or branches of the anterior part of the LCx and RCA, and with the posterior part of the LCx [1]. In 1927, Kugel described this artery as arteria anastomotica auricularis magna, which perfectly reflects its circulatory origin and morphology [2].

We present images from coronary computed tomography angiography, which documented the existence of Kugel’s artery in a 67-year-old patient with subtotal stenosis of the left main artery.

The patient was referred for coronary computed tomography angiography (CCTA) from the outpatient cardiology clinic due to hypertension and non-specific chest pain.

The CCTA examination was performed using a 384-slice dual-source SOMATOM Force computed tomography scanner (Siemens Healthcare, Erlangen, Germany). Acquisition was performed at a heart rate of 87 bpm, for the native phase at 120 kV, and for the angiographic phase at 100 kV, after intravenous administration of 65 mL of iodinated contrast agent Iomeron 400 (Bracco UK Ltd., Oxford, UK) at a rate of 5.0 mL/s. The examination was performed using retrospective ECG signal gating with dose modulation of ionizing radiation. Image reconstructions were obtained in slices 3.0 mm thick for the native phase and 0.6 mm thick for the angiographic phase. The ionizing radiation dose for the entire acquisition (total DLP) was 515 mGy·cm.

The coronary artery calcium score was 496. The risk of significant coronary artery disease was high. The calcium score of subsequent branches of coronary arteries was 88.2 for the left main (LM), 308.5 for the left anterior descending (LAD), 92.0 for the left circumflex (LCx), and 7.4 for the right coronary artery (RCA). The case was classified as A3/N4 in the CAC-DRS system, Figure 1.

The quality of the CCTA angiographic phase was assessed as good. Subtotal LM stenosis (over 90%) caused by mixed atherosclerotic plaque was observed, Figure 2.

LM was divided into three branches: LAD, LCx, and the intermediate branch (IR), Figure 3.

The presence of significant LAD stenosis was demonstrated (over 70%). LCx was co-dominant. LCx revealed insignificant stenoses (25–50%). RCA was also co-dominant. The RCA also revealed insignificant stenoses (25–50%), Figure 4.

In the CAD-RADS system, the case was classified as 4B, i.e., a case of severe CAD requiring invasive coronary angiography verification.

Additionally, an atypical connection of LCx and RCA was found at the level of the transition into the posterior descending artery (PDA), i.e., the anomalous artery of Kugel, Figure 5.

In functional images, the left ventricular ejection fraction was estimated at 50%. A three-leaflet aortic valve was imaged, without calcifications. No calcifications were found in the mitral valve.

The patient underwent further invasive coronary angiography confirming significant LM stenosis. Percutaneous coronary intervention with LM stent implantation was performed.

In most case reports published so far, Kugel’s artery was detected using invasive coronary angiography [1,3]. It has also been shown that the artery of Kugel is easy to visualize by invasive coronary angiography in vivo if a right-sided view is used for the right arteriogram and a left-sided view for the left arteriogram. In both views, the cephalic angle will often allow the arteries to be placed in the middle of the view, parallel to the AVN artery, avoiding overlap with other vessels in the atrioventricular sulcus [1].

In one study written by Nerantzis et al., the presence of Kugel’s artery was demonstrated in 6% of the subjects (6 cases/100 ex vivo angiographic studies) [1]; in 2 cases, it originated from the RCA and ended there; in 1 case, it originated in the LCx and ended there; and in 1 case, it originated from the RCA running through the sinus node artery, ending in LCx. Interestingly, in all the cases examined, the presence of an anastomotic network of small atrial branches in the lower interatrial septum was demonstrated, directly connecting the proximal and distal ends of large coronary vessels. However, the artery of Kugel provided additional direct anastomosis in the same area of the heart in six cases [1].

In the literature, there are only a few reports documenting the presence of the Kugel artery using computed tomography angiography. In the study by Saremi et al., images of the Kugel artery from coronary angiography and computed tomography angiography were presented [4]. It should be noted that the case concerned coronary artery ostial atresia, and not atherosclerotic stenosis of the coronary arteries, as in our case. The computed tomography angiography images were obtained using a 64-slice CT scanner, in suboptimal acquisition conditions; hence, they are blurry. In another study, Saremi et al. discuss the possibility of imaging small arteries of the coronary circulation using computed tomography. They presented imaging of the arterial supply to the sinoatrial node (SAN) and the atrioventricular node (AVN), which may also be the basis for the development of collateral circulation [5]. The images discussed also came from an examination using a 64-slice CT scanner. The images currently presented are from a 384-slice CT scanner and were obtained under optimal conditions. The images presented show the progress in the quality of non-invasive cardiac imaging that has occurred over the last 15 years.

The development of non-invasive imaging methods results in an increase in the usefulness and importance of coronary artery computed tomography (CCTA) in the diagnosis of coronary artery disease (CAD), as indicated by the guidelines of the European Society of Cardiology. The 2024 ESC guidelines for the management of chronic coronary syndromes indicate that CCTA is a practical, non-invasive test with proven diagnostic efficacy in detecting coronary artery stenoses. It is recommended that in people with suspected chronic coronary syndrome (CCS) and a low or moderate (>5–50%) probability of obstructive CAD before the test, CCTA is recommended for the diagnosis of obstructive CAD and to estimate the risk of major adverse cardiovascular events (MACE) (recommendation 1A). CCTA is also recommended for those with a low or moderate (>5–50%) pre-test probability of obstructive CAD to refine the diagnosis if other non-invasive tests are nondiagnostic (recommendation 1B). CCTA is not recommended for patients with severe renal impairment (eGFR < 30 mL/min/1.73 m^2^), decompensated heart failure, extensive coronary calcification, rapid or irregular heart rate, significant obesity, inability to cooperate with breath-hold commands, or any other condition that would make obtaining good image quality unlikely (recommendation 3C) [6].

The advantage of CCTA over ICA in imaging collateral arteries results from the higher spatial resolution of the method, the possibility of performing additional cross-sections in any plane and at any angle (including during the assessment after the examination, in post-processing), as well as the visualization of other anatomical structures (both cardiac and extracardiac). CCTA also provides the possibility of assessing the morphology and composition of atherosclerotic plaques causing coronary stenosis. The disadvantage of CCTA over ICA is the static and non-selective nature of the obtained image of the coronary arteries. In CCTA, the entire coronary circulation is practically simultaneously contrasted. The obtained images show the degree of contrast of the coronary arteries at one-time point [7,8,9].

Previous studies have shown that collateral circulation becomes visible when occlusion exceeds 90% [10,11]. The presence of this anastomotic communication may play a pathophysiological role in patients with dominant RCA and co-occurring coronary artery disease affecting the right coronary artery system. In patients with coronary artery occlusion, the presence of this connection bypassing the area of the occluded vessel may be a factor that prevents significant necrosis of a specific area of the myocardium.

The presence of Kugel’s artery and other collateral circulation is important when planning and performing recanalization of coronary chronic total occlusions [12,13]. The location of the origin of this artery is also significant during cardiac surgical interventions and during procedures performed by electrophysiologists performing procedures near the coronary arteries to prevent the potential risk of vascular damage [5].

The main limitation of the current article is the lack of images from invasive coronary angiography, which confirmed significant LM stenosis. It should be noted, however, that confirmation of significant LM stenosis in ICA was obtained. LM PCI was performed. Examples of Kugel artery imaging in ICA are available in the literature to date.

In conclusion, the presented images clearly indicate that coronary computed tomography angiography can visualize the existing rescue collateral circulation in patients with significant coronary artery disease.

## Figures and Tables

**Figure 1:**
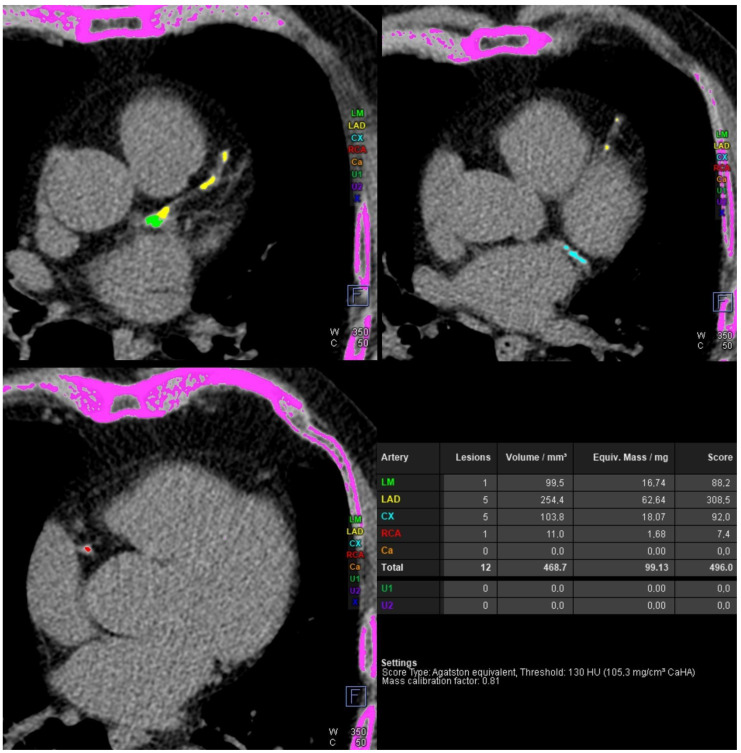
Coronary computed tomography angiography. Native phase. Coronary artery calcium score assessment. Calcifications in the left main (LM) are marked in green, the left anterior descending (LAD) in yellow, in the left circumflex (LCx) in blue, in the right coronary artery (RCA) in red and extracoronary calcifications in magenta.

**Figure 2:**
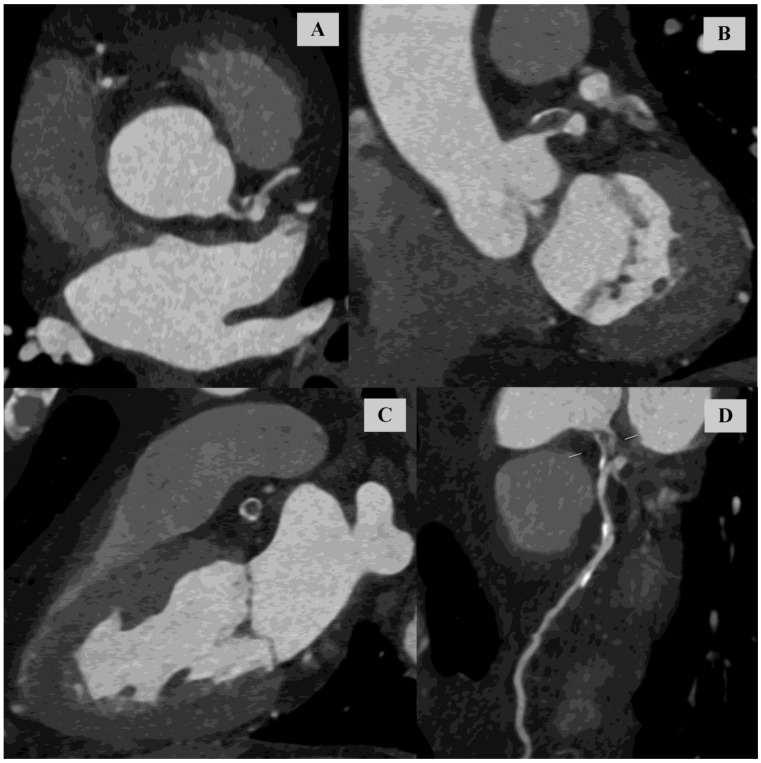
Coronary computed tomography angiography. Angiographic phase. (**A**) Multiplanar reconstruction (MPR) of the left main (LM) in a quasi-axial view, parallel to the long axis of the LM. (**B**) MPR of the LM in a quasi-coronal view, parallel to the long axis of the LM. (**C**) MPR of the LM in an oblique view, perpendicular to the long axis of the LM. (**D**) Curved multiplanar reconstruction (cMPR) of the left main (LM) extending into the left anterior descending (LAD). The gate (white dashed line) indicates the level of maximum LM stenosis.

**Figure 3:**
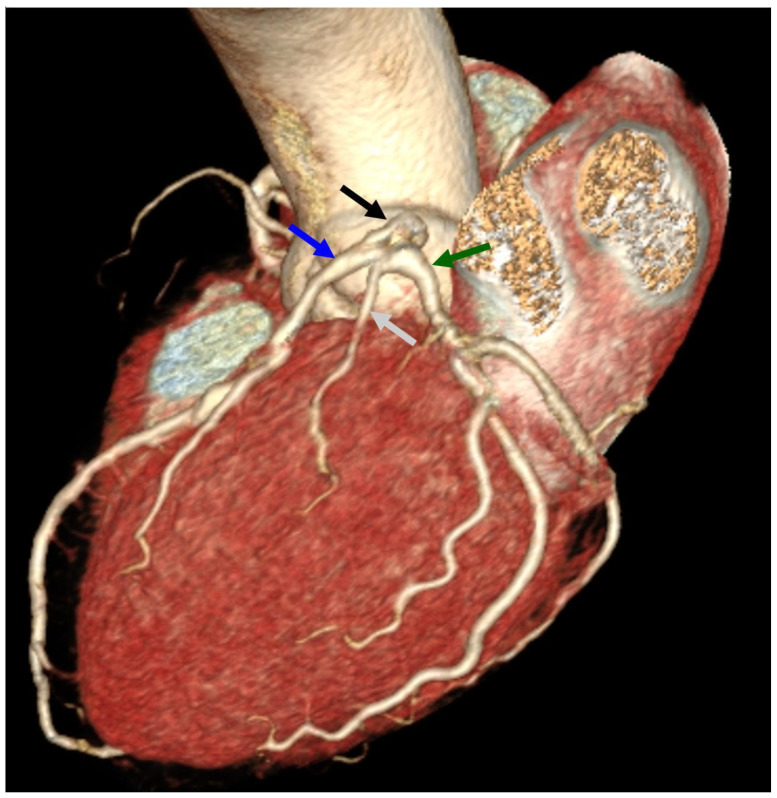
Coronary computed tomography angiography. Angiographic phase. Volume rendering reconstruction (VRT reconstruction). The black arrow indicates the left main (LM), the blue arrow indicates the left anterior descending (LAD), and the green arrow indicates the left circumflex (LCx). The gray arrow indicates the intermediate ramus (IR) resulting from the tripartite division of the LM.

**Figure 4:**
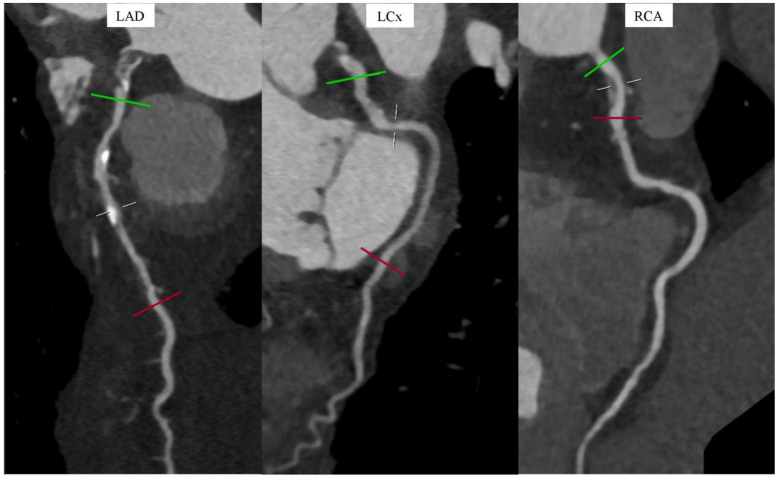
Coronary computed tomography angiography. Angiographic phase. Curved multiplanar reconstructions (cMPR) of the left anterior descending (LAD), the left circumflex (LCx), and the right coronary artery (RCA), (successive panels from the left). The green gate (solid green line) indicates the reference level of the nonstenotic arterial lumen above the level of stenosis. The red gate (solid red line) indicates such a reference level below the stenosis level. The white gate (dashed white line) indicates the level of maximum visualized coronary artery stenoses.

**Figure 5:**
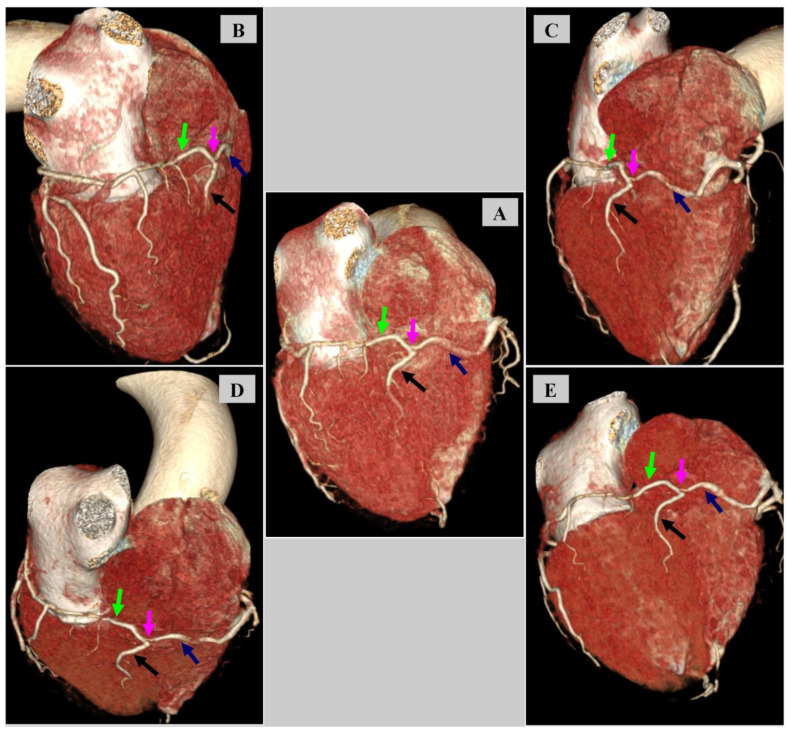
Coronary computed tomography angiography. Angiographic phase. 3D volume-rendering technique (VRT). The anomalous artery of Kugel. The green arrow indicates the left circumflex (LCx), the blue arrow indicates the right coronary artery (RCA), and the black arrow indicates the posterior descending artery (PDA). The magenta arrow indicates the Kugel artery. (**A**) Oblique view focused on the artery of Kugel. (**B**) Oblique view of the artery of Kugel from the LCx side. (**C**) Oblique view of the artery of Kugel from the RCA side. (**D**) Oblique view of the artery of Kugel from the atria side. (**E**) Oblique view of the artery of Kugel from the apical side.

## Data Availability

The original contributions presented in the study are included in the article, further inquiries can be directed to the corresponding author.

## References

[1] Nerantzis C.E., Marianou S.K., Koulouris S.N., Agapitos E.B., Papaioannou J.A., Vlahos L.J. (2004). Kugel’s artery: An anatomical and angiographic study using a new technique. Tex. Heart Inst. J..

[2] Kugel M. (1928). Anatomical studies on the coronary arteries and their branches. Arteria anastomotica auricularis magna. Am. Heart J..

[3] Soto B., Jochem W., Karp R.B., Barcia A. (1973). Angiographic anatomy of the Kugel’s artery. Am. J. Roentgenol..

[4] Saremi F., Goodman G., Wilcox A., Salibian R., Vorobiof G. (2011). Coronary artery ostial atresia: Diagnosis of conotruncal anastomotic collateral rings using CT angiography. JACC Cardiovasc. Imaging.

[5] Saremi F., Abolhoda A., Ashikyan O., Milliken J.C., Narula J., Gurudevan S.V., Kaushal K., Raney A. (2008). Arterial supply to sinuatrial and atrioventricular nodes: Imaging with multidetector CT. Radiology.

[6] Vrints C., Andreotti F., Koskinas K.C., Rossello X., Adamo M., Ainslie J., Banning A.P., Budaj A., Buechel R.R., Chiariello G.A. (2024). 2024 ESC Guidelines for the management of chronic coronary syndromes. Eur. Heart J..

[7] Xie Q., Zhou L., Li Y., Zhang R., Wei H., Ma G., Tang Y., Xiao P. (2023). Comparison of prognosis between coronary computed tomography angiography versus invasive coronary angiography for stable coronary artery disease: A systematic review and meta-analysis. Front. Cardiovasc. Med..

[8] Xie Q., Zhou L., Li Y., Zhang R., Wei H., Ma G., Tang Y., Xiao P. (2023). The ability of computed tomography angiography to predict 5-year mortality in the SYNTAX III REVOLUTION trial. J. Cardiovasc. Comput. Tomogr..

[9] Masuda S., Revaiah P.C., Kageyama S., Tsai T.-Y., Miyashita K., Tobe A., Puskas J.D., Teichgräber U., Schneider U., Doenst T. (2024). Quantitative coronary computed tomography assessment for differentiating between total occlusions and severe stenoses. J. Cardiovasc. Comput. Tomogr..

[10] Gensini G.G., da Costa B.C. (1969). The coronary collateral circulation in living man. Am. J. Cardiol..

[11] Sheldon W.C. (1969). On the significance of coronary collaterals. Am. J. Cardiol..

[12] Vo M.N., Brilakis E.S., Kass M., Ravandi A. (2015). Physiologic significance of coronary collaterals in chronic total occlusions. Can. J. Physiol. Pharmacol..

[13] Di Mario C., Mashayekhi K.A., Garbo R., Pyxaras S.A., Ciardetti N., Werner G.S. (2022). Recanalisation of coronary chronic total occlusions. EuroIntervention.

